# Pregnancy Complications in a-Thalassemia (Hemoglobinopathy H): A Case Study

**DOI:** 10.1155/2018/8532081

**Published:** 2018-05-27

**Authors:** Marianna Politou, Giorgos Dryllis, Maria Efstathopoulou, Serena Valsami, Faidra-Evangelia Triantafyllou, Athanasia Tsaroucha, Antonios Kattamis, Nikos F. Vlahos

**Affiliations:** ^1^Department of Hematology and Blood Transfusion Unit, Aretaieion Hospital, School of Medicine, National and Kapodistrian University of Athens, Greece; ^2^Anesthesiology Clinic, Aretaieion General Hospital, School of Medicine, University of Athens, National and Kapodistrian University of Athens, Greece; ^3^Second Department of Obstetrics and Gynecology, Aretaieion General Hospital, School of Medicine, National and Kapodistrian University of Athens, Greece; ^4^First Department of Pediatrics, University General Hospital “Attikon”, School of Medicine, National and Kapodistrian University of Athens, Greece

## Abstract

Thalassemia intermedia (TI) is a clinical definition which represents a wide spectrum of thalassemia genotypes but mainly includes patients who do not require or only occasionally require transfusion. An uncommon case of a 32-year-old Greek woman, para 1, at the 22nd week + day 3 of gestation with thalassemia intermedia (she was splenectomized), where her pregnancy was complicated with portal vein thrombosis, splenic thrombosis, and partial HELLP, is described. This is a generally uncommon event in thalassemia intermedia. She had no transfusion as her hematologist consulted and she took anticoagulation therapy. Thus, we present for the first time in the literature a case of HbH a-thalassemia pregnant woman whose pregnancy was complicated with portal vein thrombosis, splenic vein thrombosis, and partial HELLP; she was treated with anticoagulation therapy and she had a successful outcome.

## 1. Introduction

Thalassemias are hereditary disorders that result from the quantitative changes of alpha or beta chains of hemoglobin. Thalassemia intermedia (TI) or NTDT (Nontransfusion Dependent Thalassemia) is a clinical definition which includes patients with a wide spectrum of both alpha and beta thalassemia genotypes, who do not require lifelong regular transfusions for survival, although they may require occasional transfusions in certain clinical settings and for defined periods of time. HbH disease is the result of absence of the three *α*-globin genes (--/-*α*) due to deletional or nondeletional mutations. The phenotypic expression of the disease is wide and the clinical symptoms are a result of both hemolytic anemia and ineffective erythropoiesis [1, 2].

Progress in the management of TI patients enabled increasing rates of pregnancies among TI women [3]. Patients with TI have an increased risk of thrombosis [4] and pregnancy, as a hypercoagulable state increases that risk further. We here present a woman with TI a-thalassemia (hemoglobinopathy H), whose pregnancy was complicated with portal vein thrombosis and partial HELLP.

## 2. Case Study

A 32-year-old Greek woman, para 1, at the 22nd week + day 3 of gestation, was referred to the A&Es with a 1-week history of abdominal discomfort and persistent vomiting. The woman had HbH disease (thalassemia intermedia) with a - a 3.7/Hb Icaria a genotype. She was splenectomized at the age of 6.5 years and she maintained a Hb around 8 g/dl without blood transfusions. Her medical history included incidents of superficial-vein thrombosis in 2005, 2008, and 2009 (with negative family history of thrombosis) for which she was receiving acenocoumarol. Since the confirmation of pregnancy she was receiving Salospir 100 mg p.o. and Enoxaparin 40 mg sc.

Laboratory exams revealed moderate hypochromic microcytic anemia (Ht 28.1%, Hb 8.46 g/dL, MCV: 67.2, and MCH: 20.2), mild thrombocytosis (PLTs: 519 × 109/L), and leukocytosis: WBC 27 × 109/L (neu: 87%, lymph: 9%, and mono: 4%). The C-reactive protein was 11.4 mg/L. INR and aPTT were within normal limits but fibrinogen was high, 519.9 mg/L, and D-dimers were >5000 *μ*g/L. The rest of the biochemistry showed the following: TBil: 1.50 mg/dl; dBil: 0.8 mg/dl; SGOT 110 IU/L; SGPT 176 IU/L; ALP 165 IU/L; *γ*-GT 46 IU/L; and ferritin: 63 ng/ml, indicative of hepatic impairment. An abdominal ultrasound revealed portal vein and spleen-vein thrombosis. The patient underwent a thrombophilia testing which was negative for hereditary thrombophilia (FV Leiden, FIIG20210A) as well as for anticardiolipins antibodies. Because of the site of the thrombosis (visceral), latent myeloproliferative neoplasms (MPN) were also excluded (she was negative for the JAK2V617F mutation).

The patient was treated with enoxaparin 60 mg bd, sc, and Salospir 100 mg p.o. o.d. Transaminases were normalized two weeks later and a new abdominal ultrasound, performed two months later, showed chronic portal vein and chronic spleen-vein thrombosis.

At the 35th week + day 3, the liver enzymes and the rest of the biochemistry were as follows: SGOT 466 IU/L, SGPT 931 IU/L, ALP 220 IU/L, *γ*-GT 41 IU/L, TBil: 1.80 mg/dl, Ht 38%, Hb 10.2 g/dL, WBC 12.3 × 109/L, and PLTs: 487 × 109/L. The abdominal ultrasound showed no new findings. With the clinical suspicion of partial HELLP syndrome (the patient had only elevated liver enzymes) an urgent caesarian section (C-section) was performed. Anticoagulation bridging with heparin was used perioperatively. During the C-section, the patient developed hypertension episode [blood pressure: 190 (systolic)/ 110 (diastolic) mmHg] and she was treated with hydralazine. The premature newborn was 1810 gr but healthy and stayed in the neonatal ICU for 7 days.

Mother's liver enzymes were normalized within five days after the delivery and the patient was discharged. The patient is now on long-term anticoagulant therapy with acenocoumarol. The imaging of splenic veins is unchanged.

## 3. Discussion

The clinical manifestations of thalassemia intermedia result from ineffective erythropoiesis, chronic anemia, and iron overload. HbH which is a form of NTDT has a diverse phenotypic presentation depending on the degree of alpha globin chain deficiency which in turn relates to the underlying a-thalassemia mutations with hemolysis being the dominant clinical symptom [1]. Our patient had HbH disease and she was compound heterozygote for deletional and nondeletional mutations (–-*α* 3.7/*α*Icaria *α*). She had an intermediate phenotype that is characterized by a diagnosis at age between 2 and 4 years, maintenance of a Hb above 8.0 g/dl without transfusions, and moderate splenomegaly. She had a component of both hemolysis and ineffective erythropoiesis since ineffective erythropoiesis is more severe in nondeletional genotypes [5].

Most data in the literature concerning thrombosis in thalassemia come from b-thalassemia. Thromboembolic events occur in both b-thalassemia major and NTDT patients [6]. Thromboembolic events happen in TM and TI thalassemia patients with a frequency of 4.3%  *κα*ι 5.2%, respectively [7, 8]. The prevalence of thromboembolic events is even higher in transfusion-independent, splenectomized patients (29%), as in our case, compared to regularly transfused TM patients (2%) [9].

The underlying mechanisms that cause a hypercoagulable state in thalassemia include the oxidative damage of the red blood cell membrane proteins (due to hemolysis), endothelial impairment, platelet activation, and strong inflammatory reaction involving cytokines, adhesion molecules, and neutrophil activation. Splenectomy increases platelet counts and induces membranes abnormalities that further increase hypercoagulability [10].

In general, pregnancy in thalassemia intermedia patients can be complicated with automatic miscarriages, fetal loss, preterm delivery, IUGR, and thrombosis [11]. Oxidative stress due to iron overload and placental hypoxia caused by maternal anemia seem to provoke such complications. Pregnancy in HbH disease may be complicated mainly with anemia [12]. A study by Tongsong et al. has shown that common obstetric complications such as antepartum hemorrhage, preeclampsia, and postpartum hemorrhage are not significantly associated with HbH disease [13] but Tantiweerawong et al. have found that HbH may adversely affect maternal health [14].

However, our patient delivered at term and her fetus was not small for gestational age.

It is of interest that despite the established association of pregnancy with a hypercoagulable state, existing studies in TI pregnant women have not reported an increased risk of thromboembolic complications compared to nonpregnant TI women [3].

In our study, pregnancy was complicated with portal vein thrombosis, a generally uncommon event in NTDT. The patient underwent an extended investigation for the putative underlying cause of thrombosis that included hereditary and acquired thrombophilia tests along with tests for the exclusion of a latent myeloproliferative disorder and paroxysmal nocturnal hemoglobinemia because of the site of thrombosis (visceral). The absence of any other possible known prothrombotic factor supports the notion that thrombosis could be associated with splenectomy and the component of ineffective erythropoiesis in our patient (carrier of a-icaria mutation).

Furthermore, in this study the patient presented a partial HELLP syndrome (elevated liver enzymes). The well-known pathophysiological mechanism of HELLP syndrome is based on the release of various placental factors such as soluble vascular endothelial growth factor receptor-1 (sVEGFR-1), which then binds vascular endothelial growth factor (VEGF) and placental growth factor (PGF), causing endothelial cell and placental dysfunction by preventing them from binding to endothelial cell receptors. The result is hypertension, proteinuria, and increased platelet activation and aggregation which cause thrombotic events during pregnancy. The multiorgan microvascular injury and hepatic necrosis causing liver dysfunction further contribute to the development of HELLP.

Anemia requiring transfusion often complicates pregnancy in TI patients. However, no study has evaluated obstetric outcomes based on Hb levels in TI, and therefore the decision to transfuse should be individualized depending on maternal and fetal indications. While a significant proportion of pregnancies in TI women was complicated by IUGR in a Lebanese study, blood transfusions during pregnancy do not seem to decrease the risk of IUGR as well as other complications [15]. On the contrary, Tongsong et al. demonstrated that there are several benefits of frequent transfusions during pregnancy in HbH disease, like the prevention of growth restriction, suggesting that a strict control of hemoglobin levels could result in better outcome [13]. Data from nonthalassemic cohorts suggests that hemoglobin levels above 10 g/dL during gestation are recommended for optimal fetal growth and preclusion of preterm delivery [16]. However, targeting the 10 g/dL cutoff proved being of clinical benefit to only 78% of the pregnant NTDT patients and their fetuses in an Italian case series, while the fetuses of the other 22% suffered from IUGR [17]. There is no solid Hb cutoff for blood transfusion in pregnant TI women and the only determining factors are the cardiac function and general condition of the mother and the growth status of the fetus. Blood transfusions can increase the risk of developing alloimmune antibodies that would exacerbate any preexisting hemolytic anemia, such as thalassemia [18]. As such, our patient had no transfusion as per her obstetrician's judgment, which was primed by fetal growth and maternal cardiac status and overall well-being, and after consulting with the patient's treating hematologist.

There are no specific guidelines regarding thromboprophylaxis in pregnant TI patients. Thrombophylaxis might be essential during pregnancy and the postpartum period in cases of NTDT with splenectomy or a history of recurrent abortions. According to recent data, low-dose aspirin seems to be effective in preventing thromboembolic events during pregnancy without posing a major safety risk to mother or fetus [11]. Therefore, splenectomized women, as in our case, or those with a serum platelet count above 600 × 109/L should begin or continue taking aspirin at a dose of 75 mg/day. For splenectomized women with a platelet count above 600 × 109/L it is highly recommended to administrate low-molecular-weight heparin. We could suggest that for pregnant women receiving long-term vitamin-K antagonists before their pregnancy, like in this case, it is better to receive adjusted-dose of LMWH or a therapeutic dose of LMWH, rather than prophylactic-dose, throughout pregnancy followed by resumption of long-term anticoagulants postpartum.

## 4. Conclusion

In conclusion we present for the first time in the literature a pregnant woman with HbH disease whose pregnancy was complicated with both visceral thrombosis and partial HELLP, who was treated with anticoagulation and had a successful outcome.
